# Diversity, Equity, and Inclusion in Transplantation

**DOI:** 10.3389/ti.2024.13832

**Published:** 2024-10-09

**Authors:** Maria Irene Bellini, Chloe Balleste, Paulo N. Martins, Ifeoma Ulasi, Hannah Valantine, Luciano Potena

**Affiliations:** ^1^ Sapienza University of Rome, Rome, Italy; ^2^ University of Barcelona, Barcelona, Spain; ^3^ Donation and Transplantation Institute, Barcelona, Spain; ^4^ University of Oklahoma, Oklahoma City, OK, United States; ^5^ College of Medicine, University of Nigeria, Enugu, Nigeria; ^6^ Stanford University, Stanford, CA, United States; ^7^ IRCCS University Hospital of Bologna Sant’ Orsola Polyclinic, Bologna, Italy

**Keywords:** transplant equity, diversity and inclusion, racial disparity, gender differences, organ and tissue donation and transplantation

As a component of the European Society for Organ Transplantation (ESOT) call for action in 2022, Transplant International launched a Special Issue entitled “*Diversity, Equity, and Inclusion in Transplantation*” [1]. The call for papers focused on sex, gender, ethnic and racial disparities in transplant access, management and outcomes. Emphasis was put on the changes of policies/interventions required to address the existing inequities and the needs to build a true global access and foster a culture of diversity and inclusion in transplantation research.

With regards to sex and gender inequity, studies from United States and Nepal demonstrated barriers in the liver and kidney transplant processes, limiting the access to women from entering and completing waitlist evaluation (Giorgakis et al.; Singh Shah et al.), highlighting how they face barriers to be considered for surgery. Notably, this also reflects the disparity in the living donation process [2]: in a context where countries in Southeast Asia were reported to have the lowest rates of deceased donors, the majority of kidney living donors are women, although the highest proportion of recipients are men. To further explore the disparities in this area, a review compared the top organ donor countries, to elucidate possible interventions and establish a fair transplant process in Southeast Asia (Cowie et al.). This article provides a brilliant approach by analyzing the differences in healthcare systems and how resources and organization can impact the effectiveness of transplant programs in addition to education and cultural attitude. Within the variety of economic and developmental backgrounds, the authors identified Malaysia as one of the potential countries able to build an effective deceased donor program, recognizing the general principle that there is no “one size fits all” for organ donation systems, but that government support through financial inputs in healthcare, and therefore access to publicly funded healthcare, is fundamental for successful donation and transplantation activity.

Another interesting report dealing with a sustainable model to overcome the gender and social disparity in renal replacement therapy in Low and Middle Income Countries (Zafar and Rizvi) showed that establishing satellite centers reduces patient time and travel costs, with a model of community-government partnership, where dialysis and transplantation are integrated and offered “free of cost” to all in need.

Adequate women representation is a known unmet need in clinical science, with a documented discrepancy between the epidemiological prevalence of a disease and the rate of women enrolled in related clinical trials. Vinson and Ahmed found that in the field of kidney transplantation, women’s representation is more adequate when compared to other medical disciplines. However, they remain significantly underrepresented in research trials testing immunosuppressive drugs and surgical interventions. This finding is particularly striking in the context of the known increased risk of rejection for women, raising the hypothesis that this might also be partially related to the disparity in accessing interventional research.

Inequity in transplant access and management was described for rural and remote populations, as well as specific ethnic and caste groups, where cultural beliefs could be inherent causes of bias (Zhang and Mathur). Particular emphasis was given therefore to the proposal of eliminating race from eGFR calculations (Bellini et al.) in accessing national waitlist, a decision approved a few years ago by the OPTN Board [3], which settled in this way the tone towards a more equitable assessment of prospective transplant and donor candidates.

This remarkable change could have consequences for living kidney transplantation; a UK study (Ahmed et al.) investigated how to improve decision making from the healthcare professional’s perspective for people from diverse ethnic groups, as this precious resource remains underutilized. An education strategy seems realistic to implement the diversity of the organ donor registry, aiming to gain the support of key influencers (such as religious and community leaders, media editors, local figures) and tackle barriers negatively influencing support for organ donation in minority ethnic groups (Pradeep et al.) [4]. To this purpose, it is relevant to stress how difficult it remains to determine the impact of patient ethnicity, race or immigration background, especially in consideration of the inconsistency of how migrant and ethnic minority populations are defined in European studies [5]. This is why when analyzing such complex systems, it is recommended to consider an intersectionality approach (Nonterah) i.e., non-medical aspects of an individual’s life, as where they live, are raised, engage in recreational activities, and their vocation, to better represent the full environmental context leading to disparity in organ transplantation access, management and outcomes.

Luckily, the prevention and elimination of inequities related to patient characteristics is increasingly being recognized in transplant research. It has been reported that the demand for organs can largely be reduced if there is a sustained commitment to public health interventions and culturally competent approaches are implemented in the management of long-term conditions, taking also into consideration the demand from underrepresented minority populations, such as migrants (Grossi et al.). In this regard, the current state of the art in Italy was reviewed (Grossi et al.) and described that minority ethnic background individuals and immigrants present significantly higher rates of cardiovascular disease and endocrinological disorders, potentially leading to organ failure. Unfortunately, despite the presence of a public health system with universal healthcare coverage, non-European born residents are less likely to receive living kidney donation transplantation and more likely to have inferior long-term outcomes compared with European born individuals. These findings are not novel in general and reproduce what was already reported in other health national system realities, such as the United Kingdom [6] and United States [7].

To complete the insights into organ donation and transplantation in the immigrant population in Italy, a mention to the comparison of refusal rates showed that these were higher, especially in some non-native Italian populations countries, supporting the need for communication approaches tailored for cultural diversities, when discussing donation with families with a potential language barrier and a non-western cultural background (Grossi et al.).

It is worth remembering that the standard approaches to patient education and management are less likely to be effective with subjects from immigrant and/or ethnic minority groups, and instead tailored interventions to meet the needs of these populations remain a challenge. For instance, a report found that in abdominal transplant recipients language preference other than English was independently associated with delay to vaccination in the United States (de Crescenzo et al.). It would be therefore worth exploring alternative ways, for example, by the use of digital technology, as the reconstruction of education after the COVID-19 pandemic [8] revealed an unforeseen potential.

Could then modern technology help in pushing the boundaries of the XXI century transplant outcomes? Medical digitalization is nowadays being increasingly utilized in clinical practice, and it was suggested that blockchain technology (Anselmo et al.), defined as a peer-to-peer distributed database without centralized authority, could soon become of pivotal importance in overcoming some limitations of transplant programs. In particular, it was suggested that distribution ledger technology could affect the organ donor traffic in the black market, by providing a real integration between different national health systems with real-time auditability.

What could be the role of scientific societies, institutions and stakeholders? According to a survey by the Equity, Diversity, and Inclusion Committee of ESOT, reported as a qualitative research approach, the main areas of intervention included initiatives aiming to foster a culture of transparency in selection procedures, always considering diversity when evaluating candidates and anonymizing applications to eliminate inherent bias, using different languages in meetings and diverse panels in conferences, limiting the tenure of Council members, and promoting a bottom-up instead of a top-down organization (Pengel et al.). The recruitment of professionals from a variety of countries, backgrounds, and ethnicities or the facilitation of combining career and family life could be supported by initiatives such as access to digital learning solutions, i.e., webinars and online courses. In fact, the disparities described above could significantly hinder career development, which limits creativity and innovation by professionals from minority groups. Individuals from all backgrounds should instead have equal opportunities to enter and excel in their field and this will also promote scientific advancement and better care for the patients (Andacoglu et al.).

Our modern Society increasingly embraces the general concept of equity for all individuals, and organ donation and transplantation must follow this ethical principle and have a transparent system to assure no discrimination is carried out [9]. To achieve health equity, the same treatment options must be available to any individual affected by end-stage organ failure, regardless of sex, gender, race, ethnicity, socioeconomic backgrounds and their interplay. As the transplantation journey is a multistep process, the disparity affecting one or more phases, from clinician’s referral for evaluation to the actual moment when transplant occurs, should be explored for possible interventions to reduce the existing evidence in disparity when receiving an organ transplant. The aim of this Special Issue is to build on the promotion of healthcare and social equity worldwide, by highlighting possible areas of interventions, following what professional groups in the transplant community have identified as strategic initiatives or explicit goals in their mandates.
